# Study on Surface Permeability of Concrete under Immersion

**DOI:** 10.3390/ma7020876

**Published:** 2014-01-28

**Authors:** Jun Liu, Feng Xing, Biqin Dong, Hongyan Ma, Dong Pan

**Affiliations:** 1 Guangdong Provincial Key Laboratory of Durability for Marine Civil Engineering, College of Civil Engineering, Shenzhen University, Shenzhen 518060, China; E-Mails: liujun@szu.edu.cn (J.L.); incise@szu.edu.cn (B.D.); dpan@szu.edu.cn (D.P.); 2 Department of Civil and Environmental Engineering, the Hong Kong University of Science and Technology, Clear Water Bay, Kowloon, Hong Kong

**Keywords:** concrete, immersion, calcium hydroxide, surface permeability, pore structure

## Abstract

In this paper, concrete specimens are immersed in ultrapure water, to study the evolutions of surface permeability, pore structure and paste microstructure following the prolonging of immersion period. According to the results, after 30-day immersion, the surface permeability of concrete becomes higher as compared with the value before immersion. However, further immersion makes the surface permeability decrease, so that the value measured after 150-day immersion is only half that measured after 30-day immersion. The early increase in surface permeability should be mainly attributed to the leaching of calcium hydroxide, while the later decrease to the refinement of pore structure due to hydration. The two effects work simultaneously and compete throughout the immersion period. The proposed mechanisms get support from microscopic measurements and observations.

## 1. Introduction

Concrete is one of the most widely used construction materials in civil engineering. It is also the main material in hydraulic engineering, due to its excellent resistance to water permeation. When concrete contacts with water, the calcium hydroxide in hydrated cement paste (the binder phase in concrete) will be leached out [1,2]. Leaching of calcium hydroxide decreases the pH value of the pore solution, which may lead to the decomposition and even leaching of the main hydrates in concrete, *i.e*., calcium silicate hydrates (C-S-H). This will undoubtedly increase the porosity [3], and lower down the strength and impermeability of concrete.

Leaching is the dissolution and diffusion of calcium ions in cement paste in nature. It has been considered as one of the reasons that induce durability problems, and investigated extensively. As an intrinsic problem of concrete when immersed in water, leaching has been studied by a number of researchers [4–7]. Effects of the external environment on leaching were investigated by Maltais *et al.* [8], who found that when immersed in deionized water, calcium hydroxide and C-S-H were leached, while ettringite and gypsum could be formed when immersed in sodium sulfate solution. Kamali *et al.* [9] revealed the difference in leaching rates of cement pastes when immersed in different media. They concluded that the influencing extent of leaching in NH_4_NO_3_ solution after 19 days is 4–5 times higher than that in pure water after 114 days, and this is valid under both room temperature and high temperature (85 °C). Beside external surroundings, the leaching process is also affected by the microstructure of concrete. According to Maltais *et al.* [8], water-to-cement ratio (*w*/*c*) plays the most important role in controlling leaching. Smaller w/c results in denser microstructure, and improves the leaching resistance of concrete. When *w*/*c* is kept constant, the higher the aggregate volume fraction is, the lower the leaching rate will be.

Another process simultaneous with leaching is the hydration of cement in concrete, which makes the microstructure of concrete denser and improves the impermeability of concrete [10]. Under immersion condition, it might be the competition of leaching and hydration which determines the evolution of properties of concrete, e.g., permeability.

Environmental loads always affect the surface layer of concrete first to form a micro-environment of a single kind of corrosive ions or multiple corrosive substances, then these substances can transport into the interior of concrete [11,12]. The effective concentrations of the corrosive substances in the surface layer are the true boundary conditions for the transport of them in concrete. Therefore, studies on the transport properties of the surface layer and the cumulation of corrosive substances in the surface layer are essential for accurate modeling of the transport processes in concrete. Although transport properties/processes in bulk concrete have been investigated broadly, studies on transport properties of the surface layer of concrete can rarely be found in the literature [13]. To fill this gap, a series of studies need to be conducted. In the present preliminary study, concrete specimens are immersed in ultrapure water, and the evolution of the surface permeability of concrete under such immersion condition (combined effects of leaching and hydration) is investigated. Mechanisms behind the influence of the immersion are revealed in light of micro-scale experimental methods. Although this ultrapure water immersion condition is not representative of any service environments of concrete structures, the resulting data can be considered as the reference in a more extensive investigation [9,14], and can provide a baseline for potential models of transport processes in concrete subjected to complex environment.

## 2. Experimental Procedures

### 2.1. Materials

A CEM I Portland cement supplied by Shenzhen Haixing Onoda Cement Co. Ltd. (Shenzhen, China) was used as cementitious materials in the preparation of concrete mixture. In the cement, CaO, SiO_2_, Al_2_O_3_, Fe_2_O_3_, MgO, SO_3_ and K_2_O take mass percentages of 64.67%, 18.59%, 4.62%, 4.17%, 2.35%, 3.32% and 0.92%, respectively. The loss on ignition of the cement is 1.03%. Other materials involved in the experiments include ultrapure water with the electrical resistivity of 18.2 MΩ·cm at 25 °C, river sand with the fineness modulus of 2.61 and the apparent density of 2632 kg/m^3^ as fine aggregate, and gravel with the particle size of 5–20 mm and the apparent density of 2700 kg/m^3^ as coarse aggregate.

### 2.2. Experiments

Fresh concrete mixture was mixed according to a mix proportion in which the water-to-cement ratio equals 0.38, and the masses of water, cement, sand and gravel in 1 m^3^ of fresh concrete are 175, 461, 720 and 1079 kg, respectively. After being mixed, the mixture was casted in steel moulds with the dimensions of 100 × 100 × 100 mm^3^, and covered with plastic sheets. After 24 h, all specimens were demoulded and cured in a moisture room where the temperature and relative humidity were 20 ± 2 °C and >95% respectively. At the age of 30 days, a part of the specimens were taken out to test for permeability, while the other specimens were immersed in ultrapure water, with the water surface 13 mm higher than the upper surface of the specimens. The volume ratio of liquid to solid specimens is approximately 3 for the immersions, and no refreshment of the liquid was made during the immersion period. Permeability values were also tested after the immersion durations of 30, 60, 90, 120 and 150 days.

**Figure 1.**
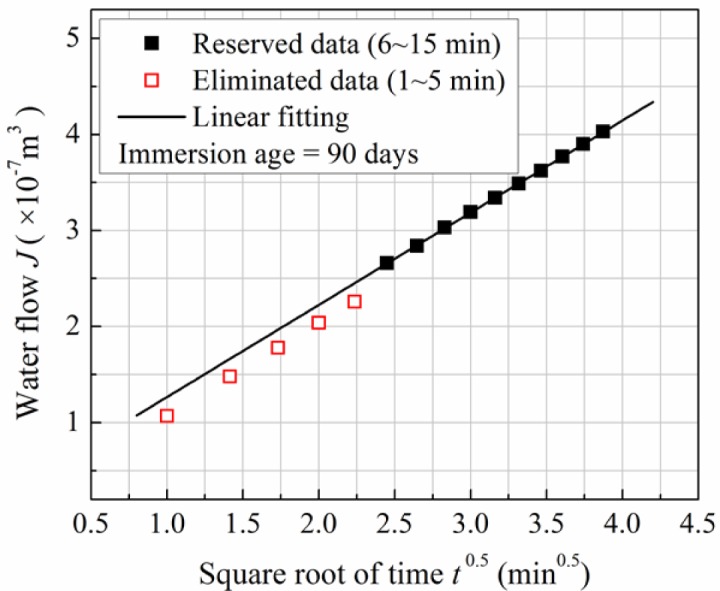
An arbitrary example of the processing of Autoclam test results.

Surface permeability was evaluated by a surface permeability index obtained from the Autoclam permeation testing equipment developed in Queen’s University Belfast, Belfast, UK. This test basically measures the volume of water penetrated in the concrete surface layer of a few millimeters, under pressure. The permeability index can be obtained from the curve of penetrated water *versus* time. The Autoclam test has been widely used to assess the permeability and other properties of normal concrete and surface treated concrete [15–17]. At the age of testing, concrete specimens were taken out, and dried in a fan assisted chamber which was maintained at 50 °C and <20% relative humidity for 1 week and then cooled overnight in ambient environment before permeability test. This kind of pre-treatment can help to eliminate the error due to the variation of moisture between samples [17]. The effective test area, which was a circle with the diameter of 50 mm, was isolated on the surface of concrete specimen using a standard steel ring and the main body of Autoclam was connected to this ring. Water was introduced to the test area and the pressure was raised to 0.5 bar (or 50 kPa) before the test was started. When the pressured water penetrated the concrete specimen tested, pressure should drop. However, more water was automatically added into the test area to maintain the test pressure of 0.5 bar. The quantity of added water was recorded along with time. The flow of water recorded could be plotted against the square root of time for the 15 min test duration, and the data points between 5 min and 15 min were used for further analysis as the points before 5 min were frequently found to be unstable. The selected data points were fitted by a line on the plot, as shown in Figure 1, and the slope of the line was referred to as the surface permeability index to compare water permeability of concrete at different immersion ages. The unit of the permeability index is normally 10^−7^ m^3^/min^0.5^, and three parallel samples were tested and averaged to get one surface permeability index.

MIP (mercury intrusion porosimetry) and nitrogen adsorption were employed to characterize the changing of the pore structure of concrete following increasing immersion ages. For MIP tests, the surface layer (within 3 mm from the surface) of concrete specimens, both before and after immersion, were sawed off and crushed, and after the elimination of large aggregate particles, small pieces were taken as samples. The samples for MIP test were dried following the solvent (ethanol) replacement drying procedure suggested by Aligizaki [18]. Based on the general suggestions in the literature [19], in the theoretical calculation of pore diameter by the well-known Washburn equation, the contact angle between mercury and hardened cement paste was chosen as 130°, while the surface tension of mercury 480 mN/m. An AutoPore IV 9500 (Micromeritics, Norcross, GA, USA) was used for MIP tests, and the maximum pressure that could be applied is 30,500 psi (210 MPa), which approximately corresponds to a minimum detectable pore diameter of 6 nm. For nitrogen adsorption, samples taken in the same way as that in MIP tests were crushed, pestled and sieved, and small particles ranging from 300 to 600 μm were collected for measurements. Nitrogen adsorption isotherms were measured by a TriSar3000 Surface Area and Pore Size Analyzer (Micromeritics, Norcross, GA, USA). Based on Kelvin equation and *t*-plot, the famous BJH method [20] was employed to deduce the pore size distributions. Powder XRD (X-ray diffraction) was employed for calcium hydroxide identification. Samples for nitrogen adsorption were crushed, manually ground with an agate mortar and a pestle, and sieved, and the powder passing the 80 μm sieve was used for XRD test. SEM (scanning electron microscopy) with EDS (energy dispersive spectrometer) was performed to observe the morphology change in immersed concrete. For this, aged concrete specimens were crushed into small pieces. After aggregate particles were eliminated, the pieces of concrete were dried under 50 °C to constant weight, and coated with a thin gold layer right before being put into the microscope.

## 3. Results

### 3.1. Surface Permeability Index

**Figure 2.**
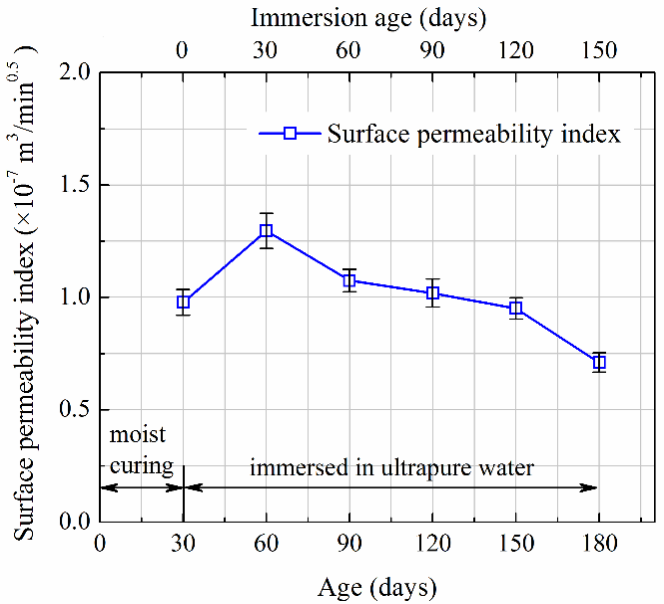
Evolution of surface permeability index of concrete as immersed in ultrapure water.

Water seepage amounts recorded within the 15 min Autoclam tests on concrete at different immersion age are listed in Table 1. According to the method as illustrated in Figure 1, the surface permeability indices can be determined. As determined, when moisture-cured to the age of 30 days, the surface permeability index of the concrete is 0.978 × 10^−7^ m^3^/min^0.5^. After immersed in ultrapure water, values of surface permeability index reach 1.296 ×10^−7^, 1.075 × 10^−7^, 1.019 × 10^−7^, 0.951 × 10^−7^ and 0.710 × 10^−7^ m^3^/min^0.5^ at the immersion ages of 30, 60, 90, 120 and 150 days, respectively. The determined surface permeability indices are plotted versus age (and immersion age) in Figure 2, and error bars showing the standard deviations are also marked at each point. It can be clearly seen that the concrete surface permeability index increases after being immersed in water, and reaches the maximum value at the immersion age of 30 days. In the first 30 days of immersion, the surface permeability index increases around 30%. Thereafter, however, the surface permeability decreases continuously, and gets back to the level right before immersion at the immersion age of 120 days. The value measured at the immersion age of 150 days is only 55% of the maximum value.

### 3.2. Pore Structure

**Figure 3.**
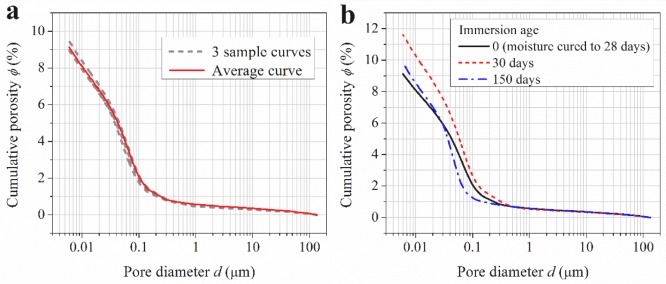
MIP cumulative porosity curves showing: (**a**) a curve averaged from three sample curves; and (**b**) changing of the pore structure of concrete as immersed in ultrapure water.

The pore size distribution (or cumulative porosity) curves of the concrete at the immersion ages of 0, 30 and 150 days, as measured by MIP, are shown in Figure 3. Figure 3a illustrates the good reproducibility of the MIP measurements, based on which three parallel measurements are averaged to get one representative cumulative porosity curve. All the three curves in Figure 3b are such average curves. It can be seen that after being immersed in water, concrete porosity increases and the pore structure gets coarser first, but after the immersion age of 30 days, porosity decreases and the pore structure becomes finer. This is consistent with the evolution trend of the surface permeability index as shown in Figure 2, which implies that higher porosity and coarser pore structure mean higher permeability.

**Figure 4.**
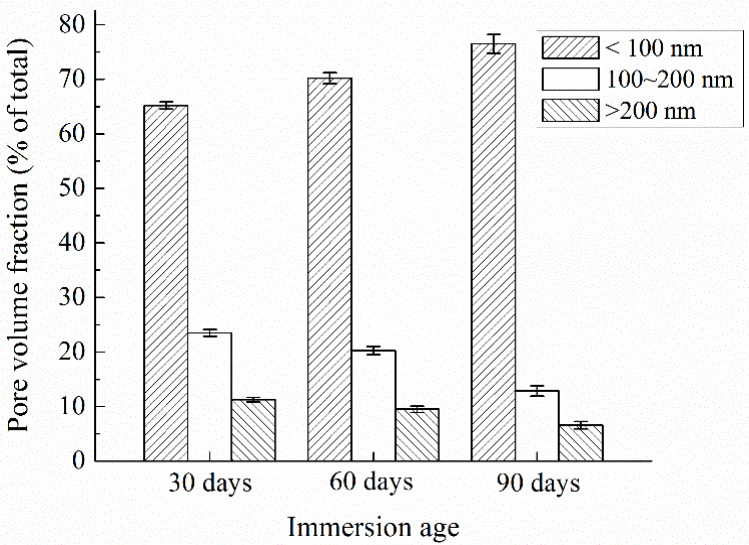
Changing of pore structure of concrete as immersed in ultrapure water (nitrogen adsorption).

Diamond, who introduced MIP into concrete technology in 1970s, has pointed out that the MIP pore size distribution curve is far from realistic, and MIP results are valuable only to provide intrudable porosities and threshold pore diameters which can serve as porosity index and connectivity index, respectively [21]. In contrast, nitrogen adsorption can realistically measure the pore size distribution in the relevant pore size range [10,18]. Pores in concrete have been classified into several types according to various criteria [22–24]. Widely accepted characteristic pore sizes include 100 nm as the lower boundary of harmful pores, and 200 nm as the lower boundary of very harmful pores. Pores smaller than 100 nm can be considered as harmless pores, and their influence on permeability can be neglected [23]. Thus, in this study, pores detected by nitrogen adsorption are classified into 3 groups, *i.e.*, <100 nm, 100–200 nm and >200 nm, and named less effective pores, effective pores and very effective pores as referring to permeability. Pore size distributions of concrete specimens at different immersion ages expressed in this way are shown in Figure 4, and the error bars in the figure show the standard deviations. Figure 4 indicates that after the immersion age of 30 days, the volume fraction of less effective pores increases while those of effective and very effective pores decrease. That is to say, the pore structure becomes finer gradually following the prolonging of the immersion period, which results in lower surface permeability index.

### 3.3. XRD and SEM

**Figure 5.**
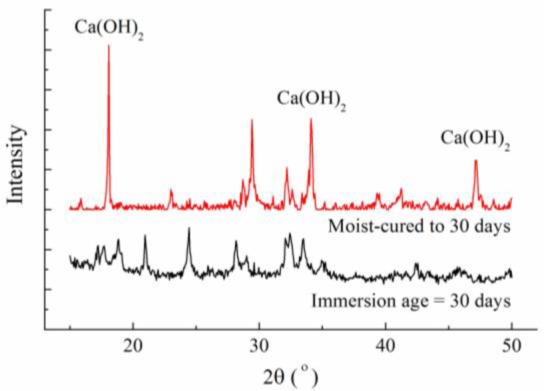
X-ray diffraction (XRD) patterns of concrete specimens at the immersion ages of 0 and 30 days.

**Figure 6.**
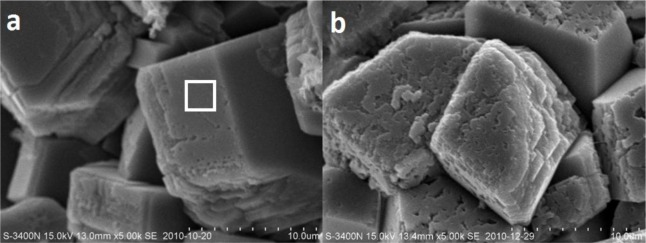
Scanning electron microscopy (SEM) images of calcium-rich crystals in concrete specimens at the immersion ages of (**a**) 0 and (**b**) 30 days.

XRD patterns of concrete specimens at the immersion ages of 0 (when concrete is moisture-cured to the age of 30 days) and 30 days are shown in Figure 5. On the patterns, peaks at 2θ of 18°, 34° and 47° indicate the existence of calcium hydroxide crystals. Through the comparison, it is clear that calcium hydroxide peaks at the immersion age of 30 days are much lower than that before immersion, and even disappear, which provides the evidence of calcium leaching. Correspondingly, SEM images taken at these two ages are shown in Figure 6. According to the result of EDS analysis performed in the white-square marked area in Figure 6a, calcium and oxide take atomic number fractions of 37.5% and 54.8%, respectively, and minor elements including carbon and silicon can also be detected. As hydrogen cannot be detected and carbon cannot be reliably detected by EDS, the deposits shown in Figure 6 can only be proven to be calcium-rich crystals, which might be partially carbonated calcium hydroxide. It can be seen that, before immersion, massive calcium-rich crystal deposits have been formed, with flat and smooth surfaces; while at the immersion age of 30 days, the surfaces of the crystals are severely influenced and become pitted. The pitted morphology could be attributed, at least partially, to calcium leaching.

## 4. Discussion

In the present study, the most interesting finding is that, as shown in Figure 2, in the first 30 days of immersion, the surface permeability index increases, but after the immersion age of 30 days, it decreases continuously. To understand this phenomenon, leaching must be considered as an essential issue. Calcium hydroxide is the most frequently discussed ingredient leached from concrete [24]. But actually, following the immersion in pure water and the leaching of calcium hydroxide, the pH value in the pore solution of the surface layer of concrete decreases somewhat, which promotes the dissolution or decalcification of even ettringite and calcium silicate hydrates [2,25,26]. In the present study, only the leaching of calcium hydroxide is traced by XRD as it is easier to be identified. Leaching normally leads to mass loss, thus lower bulk density and higher porosity [2,25], and further lower strength and impermeability. After being immersed in ultrapure water, beside calcium leaching, another issue governing the changing of concrete is the hydration of cement. Moist-cured for 1 month, the evolution rate of degree of hydration has been low. However, when immersed in water, there is sufficient water to maintain the continuous hydration of cement [10]. Thus, after being immersed, calcium leaching which leads to higher capillary porosity, and cement hydration which makes the microstructure denser, occur simultaneously and compete in the surface layer of immersed concrete specimens. This competition governs the evolution of the surface permeability index.

Since the ultrapure water was not refreshed during the immersion period, the concentration of calcium in the ultrapure water increased and the leaching rate decreased after a certain immersion period. According to Haga *et al.* [2], under the immersed condition, calcium leaching takes effect in a decelerated manner, and it is only in force in the first several weeks. In the first 30 days of ultrapure water immersion, leaching takes effects, which has been proved by Figures 5 and 6. The effect of leaching in this period could be much more dominant than that of cement hydration in changing the surface layer pore structure of the immersed concrete specimens. As a result, the overall porosity increases and the pore structure becomes coarser, as proved by comparing the MIP curves of concrete measured at the immersion ages of 0 and 30 days, as shown in Figure 3. Higher porosity and coarser pore structure mean higher permeability. This explains why the surface permeability index increases in the first month of immersion. However, following the deceleration of the leaching rate, the effect of further cement hydration may become more dominant in the two simultaneous effects. As a result, the capillary porosity decreases while the pore structure becomes finer, as proved by Figures 3 and 4, and consequently the surface permeability index also decreases with the increasing of immersion age. At the immersion age of 150 days, although the porosity is still higher than that before immersion, the pore structure has been finer, as shown in Figure 3. Under these effects, the surface permeability index of concrete at the immersion age of 150 days drops to approximately 73% of that before immersion. As compared with porosity, it seems that pore structure plays a more important role in permeability controlling.

Generally, in MIP results, a parameter named critical pore diameter (*d*_c_) can be used to assess the pore structure, *i.e.*, smaller *d*_c_ means finer pore structure. *d*_c_ can be related to permeability through the well-known Katz-Thompson equation [27].

$$\kappa =\frac{d_{c}{}^{2}}{226\cdot F}$$ (1)

where *κ* is the intrinsic permeability, and *F* is the formation factor denoting the inverse relative electrical conductivity of the target porous material. According to Yang [28], the inverse formation factor could be directly proportional to *d*_c_. In this sense, the intrinsic permeability is approximately proportional to cubic *d*_c_. In other words, the larger *d*_c_ is, the higher permeability will be. As determined from Figure 3, *d*_c_ values at the immersion ages of 0, 30 and 150 days are 73, 81 and 50 nm, respectively. This order is consistent with that of surface permeability index as shown in Figure 2.

## 5. Conclusions

Rather than transport properties of bulk concrete as focused in most researches, properties of the surface layer of concrete are emphasized in this study. The evolution of the surface permeability of concrete under immersion condition is investigated, and the mechanism behind this evolution is revealed in light of pore structure measurements, XRD and microscopic observation. Through this study, several conclusions can be drawn, as follows:

(1) After being immersed in water, calcium hydroxide will be leached from concrete, even though the concrete has been well cured.
(2) After being immersed, surface permeability of concrete increases in the first month, and then drops down from a maximum value.
(3) Leaching leads to higher porosity and coarser pore structure, while hydration makes the microstructure denser.
(4) The evolution of the surface permeability of concrete is governed by the competition of leaching and cement hydration. When leaching is dominant in the first month of immersion, the surface permeability of concrete increases. Thereafter, when cement hydration rules, the surface permeability decreases.

## Figures and Tables

**Table 1.** Water seepage volume (10^−7^ m^3^) recorded within 15 min Autoclam tests on concrete at different immersion age.

Time (min)	Immersion age (days)					
	0 (moisture curing to 28 days)	30	60	90	120	150
1	0.59	1.53	0.74	1.07	0.7	1.56
2	0.92	2.45	1.06	1.48	1.01	2.71
3	1.28	3.15	1.33	1.78	1.21	3.3
4	1.38	3.5	1.56	2.04	1.49	3.79
5	1.67	5.21	1.82	2.26	1.72	4.68
6	1.84	4.7	2.04	2.66	1.92	5.11
7	2.01	5.18	2.24	2.84	2.2	5.42
8	2.1	5.25	2.39	3.03	2.35	5.51
9	2.45	5.49	2.57	3.19	2.44	5.6
10	2.61	5.6	2.95	3.34	2.57	5.72
11	2.66	6.11	3.11	3.49	2.78	5.75
12	2.87	6.21	3.26	3.62	2.96	5.82
13	2.99	6.4	3.43	3.77	3.08	5.88
14	3.11	6.73	3.58	3.9	3.15	5.97
15	3.2	7.06	3.71	4.03	3.28	6.04
